# Draft Genome Sequences of *Vibrio fluvialis* Strains 560 and 539, Isolated from Environmental Samples

**DOI:** 10.1128/genomeA.01344-14

**Published:** 2015-01-08

**Authors:** Adonney Allan de Oliveira Veras, Miriam Lopes da Silva, Jaqueline Conceição Meireles Gomes, Larissa Maranhão Dias, Pablo Caracciolo Gomes de Sá, Jorianne Thyeska Castro Alves, Wendel Castro, Fábio Miranda, Ehilton Kazuo, Diogo Marinho, Mateus Rodrigues, Matheus Freire, Ramiro Zahlouth, Wendel Renan, Thiago Souza Lopes, Maria Helena Matté, Cintia Carolina da Silva Mayer, Suzi de Almeida Vasconcelos Barboni, Glavur Rogério Matté, Adriana Ribeiro Carneiro, Artur Silva, Rommel Thiago Jucá Ramos

**Affiliations:** a Instituto de Ciências Biológicas, Universidade Federal do Pará, Belém, Pará, Brazil; b Laboratório de Saúde Pública, Departamento de Prática de Saúde Pública, Faculdade de Saúde Pública, Universidade de São Paulo, São Paulo, Brazil; c Universidade Estadual de Feira de Santana (UEFS), Bahia, Brazil

## Abstract

*Vibrio fluvialis* is a halophilic bacterium found in many environments and is mainly associated with sporadic cases and outbreaks of gastroenteritis in humans. Here, we describe the genome sequences of environmental strains of *V. fluvialis* 560 (Vf560) and *V. fluvialis* 539 (Vf539) possessing a variant of the integrative and conjugative element (ICE) SXT for the first time in Brazil and South America.

## GENOME ANNOUNCEMENT

*Vibrio fluvialis* is a halophilic bacterium normally found in marine and estuarine environments (1). Although reports exist of the occurrence of extra intestinal cases, *V. fluvialis* is mainly associated with sporadic cases and outbreaks of gastroenteritis in humans, producing diarrhea very similar to cholera (2). Even though only two reports indicating the occurrence of diarrhea caused by *V. fluvialis* in Brazil are available in the literature, the presence of this species in the environment is well known (3, 4, 5).

The SXT, a member of the integrative and conjugative element (ICE) SXT/R391 family, was first described in clinical strains of *Vibrio cholerae* O139, the first non-O1 serogroup responsible for epidemics of cholera in the Indian subcontinent (6). Since the emergence of this element in 1992, SXT and its variants were reported among the *Gammaproteobacteria*, including *V. fluvialis* (7). Here, we describe the genome sequence of environmental strains of *V. fluvialis* 560 (Vf560) and *V. fluvialis* 539 (Vf539) that possess a variant of the ICE SXT element for the first time in Brazil and South America.

These genome sequences might help to characterize the element and enable the comparison of its structure with those already reported. Chromosomal DNA was extracted from each of the *V. fluvialis* strains by the cetyltrimethylammonium bromide method, as described by Ausubel et al. in 1995 (8).

The genome sequencing of Vf539 and Vf560 was performed using SOLiD 5500xl with a mate-paired library of 60 bp (Life Technologies) and Ion Torrent PGM (Life Technologies) with a fragment library. The SOLiD sequencing generated 267,008,850 reads for Vf539 and 263,712,284 for Vf560. Ion Torrent generated 1,776,055 reads for Vf539 and 5,440,706 reads for Vf560.

The SOLiD data were filtered with Quality Assessment software (9) using PHRED 20, leaving 199,175,010 reads for Vf539 and 188,653,932 reads for Vf560. The assembly of these data was performed with CLC Genomics Workbench software, generating, respectively, 1,336 and 972 contigs. The Ion Torrent PGM data were assembled with MIRA Assembler (10) and produced 361 contigs for Vf539 and 770 contigs for Vf560. The contigs generated by these platforms were merged and submitted to Simplifier software (11) in order to remove redundant sequences. After this step, the SSPACE (12), Gapfiller (13), CISA (14), and SeqMan pro software (DNASTAR, Inc.) were used to generate the scaffold and close gaps. At the end of the whole process, 84 scaffolds were obtained for Vf539 and 152 for Vf560, with 4,992,717 bp and 4,662,925 bp, respectively.

### Nucleotide sequence accession numbers.

The *V. fluvialis* Vf539 and *V. fluvialis* Vf560 genome sequence data have been deposited at DDBJ/EMBL/GenBank under accession numbers JQHX00000000 and JQHW00000000, respectively. The versions described in this paper are the first versions.
